# Asymptomatic *Chlamydia trachomatis* and *Neisseria gonorrhoeae* infection in men who have sex with men living with HIV

**DOI:** 10.4102/sajhivmed.v27i1.1784

**Published:** 2026-05-31

**Authors:** Remco P.H. Peters, Derek Manis, Hyunsul Jung, Lindsey de Vos, Freedom Mukomana, Buntu Mahlobisa, Renilwe Molaoa, Aphiwe Metula, Cikizwa Bongo, Oscar Radebe, Helen Struthers, Joseph Daniels

**Affiliations:** 1 Research Unit, Foundation for Professional Development, East London, South Africa; 2 Department of Medical Microbiology, Faculty of Medicine, University of Pretoria, Pretoria, South Africa; 3 Division of Medical Microbiology, Faculty of Medicine, University of Cape Town, Cape Town, South Africa; 4 Edson College of Nursing and Health Innovation, Arizona State University, Phoenix, United States; 5 Research Unit, Foundation for Professional Development, Pretoria, South Africa; 6 Wits Reproductive Health and HIV Institute, University of the Witwatersrand, Johannesburg, South Africa; 7 Research Unit, Anova Health Institute, Johannesburg, South Africa

**Keywords:** sexually transmitted Infections, STIs, men who have sex with men, MSM, HIV, intervention, South Africa

## Abstract

**Background:**

South Africa faces overlapping epidemics of HIV and sexually transmitted infections (STIs), particularly among men who have sex with men (MSM). Data on asymptomatic *Chlamydia trachomatis* and *Neisseria gonorrhoeae* infections in MSM living with HIV are limited.

**Objectives:**

To determine the prevalence and incidence of asymptomatic *C. trachomatis* and *N. gonorrhoeae* infections among MSM living with HIV.

**Method:**

We conducted a pilot randomised controlled trial of the HIV coping and disclosure management intervention among 88 MSM living with HIV in Buffalo City, South Africa. Sexually transmitted infection screening for *C. trachomatis* and *N. gonorrhoeae* was performed at baseline and at a 17-week follow-up using nucleic acid amplification testing of urine and rectal swabs. Univariable logistic regression models were used to examine the relationship between conceptually important individual-level variables and any STI at baseline and week 17 of follow-up.

**Results:**

Mean age was 30 years in the intervention arm and 33 years in the control arm. At enrolment, most participants had an undetectable HIV viral load (79.1% intervention vs 80.0% control). Syphilis positivity was higher in the intervention arm (9.3% vs 2.3%). Baseline prevalence of STIs was high, including urethral and rectal *C. trachomatis* and *N. gonorrhoeae* infections. At week 17, STI prevalence was similar between arms (27.9% intervention vs 28.9% control). Univariable analyses did not identify any factors associated with STI at follow-up in either group.

**Conclusion:**

Asymptomatic STIs are highly prevalent and incident among MSM living with HIV in South Africa. Findings highlight the need to strengthen STI prevention, treatment and care services, and to innovative interventions.

**What this study adds:** This study provides prospective data on asymptomatic *C. trachomatis* and *N. gonorrhoeae* among MSM living with HIV in South Africa, demonstrating high prevalence, and underscoring the need for routine, comprehensive STI screening strategies.

## Introduction

South Africa has overlapping high burdens of HIV and sexually transmitted infections (STIs). Higher rates of STI have been reported among people living with HIV (PLHIV) because of biological and epidemiological synergy.^1^ For example, men who have sex with men (MSM) with rectal *Chlamydia trachomatis* or *Neisseria gonorrhoeae* infection are 2 to 3 times more likely to acquire HIV infection compared to those without a rectal STI.^2^

Syndromic management is the standard of care for STIs in South Africa.^3^ This approach means that empirical antibiotic treatment is provided without diagnostic testing to individuals presenting with STI-associated symptoms such as genital discharge. Syndromic management is a relatively cheap approach that is easy to implement but also results in excessive and unnecessary use of antibiotics. Importantly, asymptomatic STIs remain untreated without diagnostic tests. A study from Cape Town showed that 91% of *C. trachomatis* and 67% of *N. gonorrhoeae* infections in MSM visiting sexual health services were asymptomatic, especially in the case of rectal infection.^4^ The higher frequency of asymptomatic manifestation of rectal infection in MSM is illustrated in the consistently higher rates of rectal than urethral infection in prevalence studies in South Africa.^5,6,7,8,9,10,11^

To date, several studies have performed molecular STI testing of MSM in South Africa, but these are heterogeneous and have relatively small sample sizes. There are four studies on the aetiology of urethral and rectal discharge; six studies (including ours) report on the prevalence of *C. trachomatis* and *N. gonorrhoeae* in asymptomatic MSM, and only two studies have reported STI incidence data.^4,5,6,7,8,9,10,11^ The anatomic sites tested for STI also vary, while two studies only reported aggregated data. Additional data points, and a comprehensive overview of the burden of STIs in MSM in South Africa, are warranted.

We recently conducted a pilot randomised-controlled clinical trial of a skills-based HIV serostatus coping and disclosure management intervention for MSM in the Buffalo City Metropolitan Municipality in East London.^12^ Participating MSM were also tested for *C. trachomatis* and *N. gonorrhoeae* at their baseline and follow-up visits, providing the opportunity to document the burden of STIs in this population. Therefore, we report on the incidence and prevalence of *C. trachomatis* and *N. gonorrhoeae* in MSM living with HIV infection in our pilot trial.

## Research methods and design

### Study design, setting and participants

We conducted a pilot randomised-controlled trial of the Speaking Out and Allying Relationships (SOAR) intervention to improve antiretroviral therapy (ART) adherence for gay, bisexual, and men who have with sex with men (GBMSM) living with HIV in the Buffalo City Metropolitan Municipality, South Africa.^13^ Specifically, SOAR consists of five one-hour group sessions delivered weekly via videoconference by a trained facilitator.^12,13^ The sessions aim to build coping skills, support disclosure decision-making, promote safer sex practices, and develop ART adherence and communication plans.^12,13^

Gay, bisexual, and men who have with sex with men were recruited through venue- and snowball sampling approaches in close partnership with Engage Men’s Health, an HIV service organisation for GBMSM in this setting. Among those interested in learning more about the study, a two-step screening and consent process was completed. First, eligibility was confirmed using a short questionnaire and HIV rapid test by the study team to confirm relationship and HIV seropositive status. If individuals were HIV positive, missed four or more doses of ART in the last 30 days, and were in a relationship for 30 days or more, then they were eligible to participate in the study.

After screening, interested individuals completed written informed consent and then enrolled. Block randomisation was applied through sealed envelopes (ensuring the study was blinded) to allocate participants to either receive the SOAR intervention or the standard of care, that is, health information, condoms, and lubrication. The SOAR intervention was 16 weeks, with follow-up visits scheduled at week 17 after randomisation at the baseline visit.

### Study and laboratory procedures

Participants completed a questionnaire with demographic questions, and a physical examination and syphilis screening using a rapid diagnostic test (Alere Determine TP test; Abbott Laboratories, Inc., Abbott Park, Illinois, United States) was performed. In case of a positive syphilis screening test, blood was drawn for further syphilis serology in the laboratory (PathCare, East London).

At the baseline and the 17-week follow-up visit, first-void urine specimens and rectal swabs (Copan Diagnostics, Brescia, Italy) were collected by the healthcare worker for *C. trachomatis* and *N. gonorrhoeae* testing. Samples were transported from East London once per week to the Department of Medical Microbiology at the University of Pretoria for further microbiological processing. DNA was extracted from specimens with the High Pure PCR Template Preparation Kit (Roche Diagnostics GmbH, Mannheim, Germany) followed by specific detection of *C. trachomatis* and *N. gonorrhoeae* DNA using the LightMix^®^ Kit *Neisseria gonorrhoeae* and *Chlamydia trachomatis* (TIB MOLBIOL Syntheselabor GmbH, Berlin, Germany) as per the manufacturer’s instructions. Participants were contacted telephonically about their STI results; in the case of a positive result, treatment was organised for them as per national STI management guidelines, namely azithromycin 1 g single dose for *C. trachomatis*, and combination therapy of ceftriaxone 250 mg plus azithromycin 1 g in the case of *N. gonorrhoeae*.^14^

### Measures

The outcome of interest was whether there was any STI outcome at the end of week 17. This was measured as a binary variable for either *C. trachomatis* or *N. gonorrhoeae*. The exposures of interest were intervention (vs control), age, education level, employment status, sexual orientation, and relationship. These were considered conceptually important variables.

Other measures that were obtained from participants pertained to anal sex, non-use of condoms, sex and alcohol, previous STI tests, and past STI diagnoses.

### Statistical analysis

Questionnaire data were directly captured in RedCAP software with checks in place for consistency and range. Laboratory results were double-entered and verified. The RedCAP database was finalised and used for analysis.

Our total sample for the analysis was 88 participants. Seventeen participants were removed because of missing data on conceptually important variables; we restricted our analysis to those who were men and were homosexual, bisexual, or pansexual. Data were presented as counts and proportions for categorical variables and median with range for continuous variables. To assess potential public health impact, we explored the count of new and existing cases and divided these by the total person follow-up time to obtain incidence and prevalence rates using the entire dataset of 105 participants. Univariable logistic regression models were used to examine the relationship between conceptually important individual-level variables and any STI infection at week 17 of follow-up. Statistical analyses, data cleaning, and data visualisation were conducted in R version 4.4.0 (R Foundation for Statistical Computing, Vienna, Austria).

### Ethical considerations

This study has been reviewed and approved by the University of Pretoria Review Board (189/2022) with reliance by the Institutional Review Board at Arizona State University (STUDY00014539).

## Results

The mean age was 30 years in the intervention arm, and 33 years in the control arm. Most participants were in some form of relationship, and the majority of participants had been tested for an STI in the last year. Nearly all participants identified as homosexual or gay (Table 1).

**TABLE 1:** Baseline characteristics of participants (*N* = 88).

Variables	Control (*n* = 45)				Intervention (*n* = 43)			
	Mean	s.d.	*n*	%	Mean	s.d.	*n*	%
Age (years)	32.8	9.42	-	-	29.7	6.79	-	-
**Sexual orientation**								
Homosexual	-	-	37	82.2	-	-	39	90.7
Bisexual	-	-	6	13.3	-	-	4	9.3
Pansexual	-	-	2	4.4	-	-	0	0.0
**Relationship status**								
Monogamous relationship	-	-	25	55.6	-	-	28	65.1
Polyamorous relationship	-	-	1	2.2	-	-	3	7.0
Non-monogamous or open relationship	-	-	7	15.6	-	-	2	4.7
Sex buddies or It’s just sex	-	-	7	15.6	-	-	6	14.0
Friends with benefits	-	-	5	11.1	-	-	4	9.3
No anal sex	-	-	3	6.8	-	-	1	2.3
Anal sex partners	7.2	4.50	-	-	8.6	11.33	-	-
Anal sex bareback partners	2.6	3.63	-	-	4.0	7.98	-	-
Drunk bareback sex partners	2.0	3.05	-	-	5.0	12.21	-	-
**Last STI test**								
< 3 months	-	-	18	40.0	-	-	13	32.5
3–6 months	-	-	7	15.6	-	-	8	20.0
6–12 months	-	-	7	15.6	-	-	13	32.5
1–3 years	-	-	4	8.9	-	-	4	10.0
> 3 years	-	-	4	8.9	-	-	1	2.5
Never tested	-	-	5	11.1	-	-	1	2.5
**STI in the last 12 months**								
Chlamydia	-	-	3	25.0	-	-	8	40.0
Gonorrhoea	-	-	2	16.7	-	-	6	30.0
Syphilis	-	-	6	50.0	-	-	4	20.0
Genital warts or HPV	-	-	1	8.3	-	-	1	5.0
Genital herpes	-	-	0	0.0	-	-	1	5.0
Hepatitis A	-	-	0	0.0	-	-	0	0.0

s.d., standard deviation; STI, sexually transmitted infection; HPV, human papillomavirus.

At enrolment, 79.1% in the intervention arm had an undetectable HIV viral load (≤ 200 copies/mL), and 80% in the control arm. Syphilis rapid diagnostic test was positive in 9.3% of the intervention arm, and 2.3% of the control arm. Treponemal antibodies were confirmed by laboratory testing and all rapid plasma reagin (RPR) tests were positive, with titres of less than 1:8.

**FIGURE 1:**
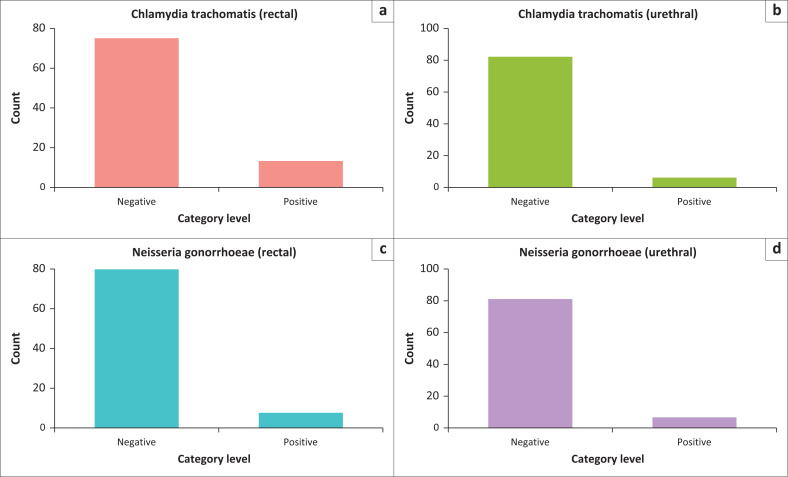
Frequencies of (a) *Chlamydia trachomatis* (rectal), (b) *Chlamydia trachomatis* (urethral), (c) *Neisseria gonorrhoeae* (rectal), (d) *Neisseria gonorrhoeae* (urethral). *Chlamydia trachomatis* and *Neisseria gonorrhoeae*.

**FIGURE 2:**
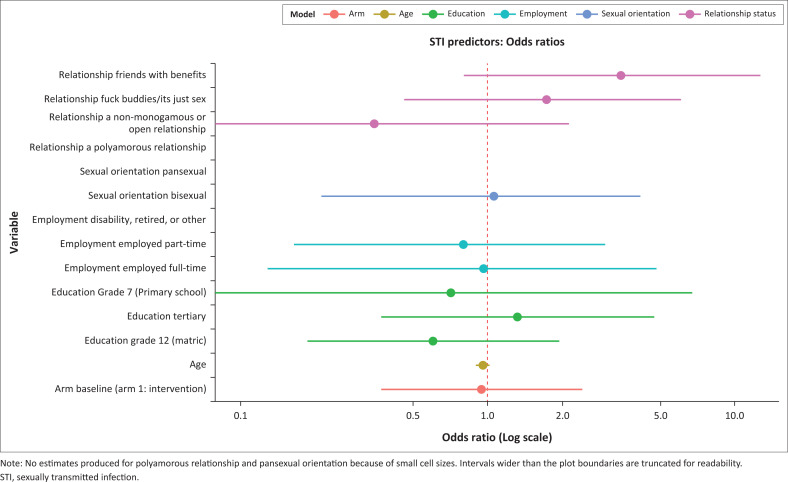
Univariable regression results.

At baseline, 82 (93.2%) had a urethral *C. trachomatis* infection; 81 (92.0%) had a urethral *N. gonorrhoeae* infection; 75 (85.2%) had a rectal *C. trachomatis* infection; and 80 (90.9%) had a rectal *N. gonorrhoeae* infection (Figure 1). At week 17, 12 (27.9%) in the intervention arm had an STI, and 13 (28.9%) in the control arm. Univariable analyses did not identify factors that were associated with any STI at week 17 (Figure 2).

Median time of follow-up was 140 days (20 weeks; range 80–304 days). Incidence of any STI was 72 per 100 person-years (95% confidence interval [CI] 50–101), with incidence of 48 per 100 person-years (95%CI 30–73) for urethral infection, and 45 per 100 person-years (95%CI 28–70) for rectal infection (Table 2).

**TABLE 2:** Incidence of sexually transmitted infections in 100 men who have sex with men in the Eastern Cape province, South Africa, 2022–2023.

Sexually transmitted infection	Incidence	95%CI (per 100 person/year)
**Any STI**	71	41–99
*Chlamydia trachomatis*	69	47–97
*Neisseria gonorrhoeae*	11	3.5–25
**Urethral infection**	47	30–71
*Chlamydia trachomatis*	47	30–71
*Neisseria gonorrhoeae*	0	-
**Rectal infection**	45	27–69
*Chlamydia trachomatis*	43	26–66
*Neisseria gonorrhoeae*	11	3.5–25

CI, confidence interval.

## Discussion

This study adds important information on the epidemic profile of STIs among MSM living with HIV in South Africa. Our findings demonstrate a high prevalence and incidence of *C. trachomatis* and *N. gonorrhoeae* infections, particularly at rectal sites, consistent with previous studies.^1,2,5,6,7,9^ Notably, 29% of participants had an asymptomatic STI at baseline, increasing to 33% at follow-up, with rectal infections more common than urethral infections. Diagnostic STI screening may provide a tool to address this burden of infection, but important implementation questions have to be addressed, including the optimal frequency of screening and the uncertain impact on population prevalence and antimicrobial resistance.

The incidence rates observed are among the highest reported in South African MSM cohorts. These observations highlight the need for strengthening STI prevention, diagnosis and treatment services, and the implementation of innovative interventions. Doxycycline post-exposure prophylaxis (DoxyPEP) may provide such an option with strong reduction in STIs reported in clinical trials as well as STI programmes, particularly for chlamydia and syphilis.^15,16,17^

Previous studies on *C. trachomatis* and *N. gonorrhoeae* from South Africa have found that urethral and rectal infections are prevalent, independent of asymptomatic infection.^5,6,7,9,10^ Our study underscores the importance of STI prevention approaches, such as DoxyPEP. Moreover, our study demonstrates a modest reduction in any STI infection among the intervention arm, which suggests the importance of upscaling the SOAR intervention for this population throughout South Africa.

### Strengths and limitations

Our study is strengthened by its prospective design, high retention rate, and integration within a randomised-controlled trial. However, limitations include the relatively small sample size and geographic restriction to Buffalo City, which may limit generalisability. Despite these limitations, our findings contribute valuable insights into STI dynamics in MSM living with HIV in South Africa, and support the need for enhanced surveillance, targeted prevention, diagnostic and treatment interventions, and policy reform.

## Conclusion

Asymptomatic STIs are highly prevalent and incident among MSM living with HIV in South Africa, with rectal infections predominating. Tailored interventions are essential to reduce transmission, improve sexual health outcomes, and align STI management with global best practices.
